# A Conceptual Study on Characterizing the Complexity of Nutritional Interventions for Malnourished Older Adults in Hospital Settings: An Umbrella Review Approach

**DOI:** 10.3390/healthcare12070765

**Published:** 2024-03-31

**Authors:** Alvin Wong, Yingxiao Huang, Merrilyn D. Banks, P. Marcin Sowa, Judy D. Bauer

**Affiliations:** 1 Department of Dietetics, Changi General Hospital, 2 Simei Street 3, Singapore 529889, Singapore; 2 School of Human Movement and Nutrition Sciences, University of Queensland, St. Lucia, QLD 4072, Australia; 3 Centre for the Business and Economics of Health, The University of Queensland, St. Lucia, QLD 4067, Australia; 4 Department of Nutrition and Dietetics, Royal Brisbane and Women’s Hospital, Herston, QLD 4029, Australia; 5 Department of Nutrition, Dietetics and Food, Monash University, Notting Hill, VIC 3168, Australia

**Keywords:** malnutrition, complex interventions, hospitalization, individualized nutrition

## Abstract

Introduction: Malnutrition is a widespread and intricate issue among hospitalized adults, necessitating a wide variety of nutritional strategies to address its root causes and repercussions. The primary objective of this study is to systematically categorize nutritional interventions into simple or complex, based on their resource allocation, strategies employed, and predictors of intervention complexity in the context of adult malnutrition in hospital settings. Methods: A conceptual evaluation of 100 nutritional intervention studies for adult malnutrition was conducted based on data from a recent umbrella review (patient population of mean age > 60 years). The complexity of interventions was categorized using the Medical Research Council 2021 Framework for Complex Interventions. A logistic regression analysis was employed to recognize variables predicting the complexity of interventions. Results: Interventions were divided into three principal categories: education and training (ET), exogenous nutrient provision (EN), and environment and services (ES). Most interventions (66%) addressed two or more of these areas. A majority of interventions were delivered in a hospital (n = 75) or a hospital-to-community setting (n = 25), with 64 studies being classified as complex interventions. The logistic regression analysis revealed three variables associated with intervention complexity: the number of strategies utilized, the targeted areas, and the involvement of healthcare professionals. Complex interventions were more likely to be tailored to individual needs and engage multiple healthcare providers. Conclusions: The study underlines the importance of considering intervention complexity in addressing adult malnutrition. Findings advocate for a comprehensive approach to characterizing and evaluating nutritional interventions in future research. Subsequent investigations should explore optimal balances between intervention complexity and resource allocation, and assess the effectiveness of complex interventions across various settings, while considering novel approaches like telehealth.

## 1. Introduction

Malnutrition is a complex disease, and its prevention and treatment require early identification and intervention [1,2]. The ESPEN 2017 guidelines [3] on definitions and terminology of clinical nutrition define nutrition therapy as “how nutrients are provided to treat any nutritional-related condition. Nutrition or nutrients can be provided orally (regular diet, therapeutic diet, e.g., fortified food, oral nutritional supplements), via enteral tube-feeding or as parenteral nutrition to prevent or treat malnutrition in an individualized way” [Strong Consensus, 97% agreement] [3].

In clinical practice and research, nutrition intervention may involve a single-component strategy such as direct provision of nutrients via the use of medical and non-medical food. More frequently, interventions involve multiple strategies, such as targeting various aspects of biochemical and biologic pathways (e.g., anti-inflammatory, appetite, and muscle stimulation) [4,5,6,7,8], nutritional knowledge (e.g., dietary counseling of patients and caregivers, and improving nutritional knowledge of healthcare professionals) [9,10,11], and service provision/quality (modifying the hospital environment, food services, and optimizing nursing and post-discharge care) [12,13,14,15,16,17,18,19].

Recently published systematic reviews and meta-analyses (SRMAs) regarding the outcomes of nutrition intervention reported highly heterogeneous results [10,12,20,21], leading to a very low certainty of evidence for the use of nutritional interventions [22], even though findings may be statistically significant. The inconsistency can be attributed to the variable study populations or the type and length of interventions included. Even within SRMAs [20,21] performed and updated by the same research groups, there exist no clear definitions to distinguish between individualized nutrition support [20] from dietitian-led intervention [21].

This methodological heterogeneity has also been observed for cachexia, sarcopenia, and muscle-related nutritional interventions [23]. While analyses based on patient populations or studies with simple linear pathways (e.g., modular macronutrient supplementation with glucose to increase energy intake) will reduce such heterogeneity, such interventions have not been shown to improve clinical outcomes. Furthermore, interactions are known to exist between various components or strategies in a complex intervention [24]. In response, the United Kingdom’s Medical Research Council and the National Institute for Health Research introduced a framework in 2021 for crafting and assessing complex interventions [24], aiming to bring clarity and structure to this field.

Transitioning from the broad perspectives provided by SRMAs to a more focused approach, the Medical Research Council framework [24] offers a structured methodology for evaluating the complexity of nutritional interventions. While SRMAs often describe and present the methodologies of nutritional interventions, important features such as the individualization of intervention strategies, complexity of interventions, qualifications of the educator, delivery method of education (e.g., face-to-face, telephone, or internet), and utilization of healthcare resources (human and financial resources, e.g., time spent in intervention by clinicians) are frequently absent, as shown in a recent review [11]. Despite the acknowledged diversity of nutritional interventions for malnutrition in hospitalized adults and elderly populations, a systematic approach to categorize these interventions based on their complexity remains unexplored. This study seeks to address the research question: “How can nutritional interventions for malnutrition in hospitalized adults be systematically categorized based on their complexity”? We hypothesize that applying an umbrella review approach will reveal distinct categories of interventions, facilitating a better understanding and implementation of nutritional therapies.

Therefore, the primary objective of this study is to systematically categorize nutritional interventions into simple or complex, based on their resource allocation, strategies employed, and predictors of intervention complexity in the context of adult malnutrition in hospital settings.

## 2. Methods

### 2.1. Data Selection

We assessed 120 primary studies from 19 systematic reviews and meta-analyses identified in a recent umbrella review (a systematic review of systematic reviews) [25] to select nutritional therapies for the prevention or treatment of adult malnutrition in hospital settings. The study designs of the primary studies included randomized controlled trials (RCTs), non-randomized clinical trials, and observational studies. The methodology for the umbrella review, including the Population, Intervention, Control, Outcomes, and Study type (PICOS), along with the details on how it was conducted, have been previously published [25]. In summary, the umbrella review evaluated SRMAs of older adult inpatient populations (mean age > 60 years for the majority of the SRMAs) and explored nutrition interventions to improve oral nutritional intake beyond standard care to determine the effectiveness of nutritional interventions on key clinical outcomes (mortality, morbidity, hospital stay length, readmissions, quality of life, and functional status). Details of the SRMAs and the primary studies are available in the Supplementary Material.

The exclusion criteria, reported in the original umbrella review [25], consisted of patient populations (1) that require highly specialized care (such as critically ill, oncology, and palliative care), (2) from developing countries, as outcomes may be systematically different from developed settings, and (3) that primarily included parenteral and enteral nutrition support, as these are life-sustaining interventions. Nutritional interventions for specific disease management (e.g., cancer cachexia, diabetes, genetic diseases, inflammatory bowel diseases, and organ failure/transplantation) were excluded as they may require non-standard experimental healthcare resources. Finally, interventions as part of a protocolized clinical treatments such as enhanced recovery after surgery (ERAS) were excluded.

### 2.2. Data Extraction and Statistical Analysis

Data were extracted for all health resources (financial and human) used in the primary study (both clinical trials and observational studies), the registered or published study protocol, and post hoc analyses of the primary publication. Intervention characteristics and strategies used in each primary study were also identified, with a qualitative and quantitative synthesis performed, guided by the Preferred Reporting Items for Systematic Reviews and Meta-Analyses (PRISMA) recommendations [26], as reported in the original umbrella review [25]. The extracted data were entered into a spreadsheet (Microsoft Excel) by AW and checked by YH. Any inconsistencies of data extraction and disagreements on categorizing resources were reviewed by a third author (JDB).

The following categorical variables were investigated: intervention type/novel strategies, primary outcomes of the study, setting (where the intervention was delivered, i.e., at home, in the community, or both), delivery method (e.g., face-to-face in hospital or clinics, written or printed materials, or via telehealth e.g., phone or video calls), individualization or standardization of intervention (adaptation of the intervention to the individual based on nutritional assessment, dietary education and adjustment, or nutritional supplementation modification by the trained clinician), intervention target (patient, policy, or the environment), and type of healthcare professional performing the intervention. These variables were derived using the human and financial resources reported in the research methods, and the following continuous variables were computed: frequency and duration of the intervention, and the number of unique strategies and healthcare professionals involved.

### 2.3. Health Resources in Nutritional Interventions

#### 2.3.1. Financial Resources or Health Spending

A measurement of health services and goods consumption, which includes health facilities such as inpatient (admissions classified by diagnostic-related group or hospital bed days classified by the intensity of ward care), outpatient, sub-acute, and long-term care, as well as public health and administrative services. This also includes consumables such as pharmaceutical, medical, and nutritional products [25]. Commonly recorded resources include visits to the doctor, nurse, or allied health professional, and medications administered (by dosage, frequency, and route of administration). These may be recorded as one-off events or aggregated over a time period (month or year). We assessed the health spending based on the study intervention and follow-up periods to determine the overall use.

#### 2.3.2. Human Resources

These include medical, nursing, and allied health professionals who are involved in the delivery of health services [25]. For this study, we also included ancillary non-clinical staff and volunteers as human resources, as these individuals are involved in supportive or assistive interventions, such as meal ordering, food services, feeding assistance, and delivery of medications and medical food.

#### 2.3.3. Resources Excluded

Research-related resources not representative of routine care (e.g., study visits by research coordinators, clinic visits specific for study reviews, and non-standard care in specialist centers) were excluded unless they were essential components of nutritional care. Essential nutritional care includes home visits for nutritional assessment and management, delivery of medical food and meals, and subscription of services such as meals on wheels. While money gives command over resources, it is not a resource per se, and hence any gratuities in the form of cash or gift to participants, volunteers, or staff from the studies were excluded.

Resources may be grouped together under one category to reduce the number of variables where appropriate, for example individual vitamin or mineral supplements, and multivitamins with or without minerals, under the family of “micronutrients” if the use is rare or infrequent.

***Classification of Nutritional Intervention into Simple or Complex Intervention***

The Medical Research Council 2021 updated guidance provides a framework for developing and evaluating complex interventions [24], and was used to evaluate if a nutritional intervention is simple or complex. In this framework, complex interventions are commonly described as interventions that contain several interacting components, and show characteristics of emergence, feedback, adaptation, and self-organization [24].

Other considerations of a complex intervention include the presence of a synergistic relationship between the interacting components; the presence of mediating and/or moderating factors that assert an effect on the intervention; possible susceptibility of the intervention to the effect of different contexts such as policy timing, organizational culture, and leadership; health resources allocation; staffing levels and capabilities; interpersonal relationships; and non-linear relationships of input and output [25]. The criteria are presented and summarized in Table 1. One author (AW) applied the framework to each of the primary studies to identify simple and complex interventions, and a second author (YX) reviewed the classification. Any disagreements on classification were reviewed by the three other authors (MDB, MPS, and JDB).

***Individualization versus Standardization of Nutritional Interventions***

Additionally, interventions were identified as individualized if the assessment of nutritional requirements was performed using indirect calorimetry or validated predictive equations and one of the following interventions:

(1) Dietary education or counseling by healthcare workers specializing in clinical nutrition to patients or caregivers, to meet or increase energy/ protein goals, and tailored to individuals’ habitual intake or preferences.
(2) Supplementing intake with medical and non-medical food, with or without micronutrients (multivitamins or minerals and trace elements), on admission to hospital or adjusted during the intervention period, and where intervention is tailored to meet the patient’s requirement and needs (flavor, variety, and/or nutrient content).

Otherwise, the intervention was classified as standardized, which includes provision of standard nutrition education pamphlets or general nutritional advice for healthy eating and adequate intake, or using fixed dosages of oral nutritional supplements (e.g., 2 bottles per day) for all patients.

### 2.4. Statistical Analysis

Categorical variables are reported as counts and percentages. The Shapiro–Wilk normality test determined that all the continuous variables were not normally distributed, and are therefore reported as medians with interquartile ranges (IQRs). Categorical variables were compared using χ^2^ and Fisher exact tests, and the Mann–Whitney test was used for continuous variables for selected groups. Binary logistic regression analyses were conducted to identify the factors associated with the complexity of nutritional interventions. Covariates for the models were selected based on relevance, which included human and financial resources, types of nutritional strategies used, individualization or standardization of interventions, and the computed continuous variables for the number of interventions and healthcare professionals involved. In the multivariate logistic regression models, each variable was adjusted for covariates that were associated with the variable on univariate analyses. A *p*-value of 0.1 (α = 0.1) was used as the cut-off for independent variables for the model.

Multicollinearity was assessed using the variance inflation factor, with a factor exceeding 5 indicating high multicollinearity between the independent variable and the other variables [25]. Nonlinearity was assessed using the residual plot of fitted values compared to the residuals, and by testing quadratic terms of the continuous variables in the models. Regression analysis results are reported as Exp β and their corresponding 95% Wald Confidence Interval (odds ratio [OR] > 1.0 suggesting greater association of the covariate with the outcome). Sensitivity analysis was performed for interventions that targeted individual patients. Two-sided significance testing was used, and *p* < 0.05 was considered statistically significant without adjustment for multiple testing. Sensitivity analysis was also performed for nutritional interventions originating from RCTs only. All analyses were performed with the open source program JASP (version 0.16.4, Apple Silicon, University of Amsterdam, Amsterdam, The Netherlands) [27].

## 3. Results

### 3.1. Description and Features of Nutritional Interventions and Strategies

We included 100 of 120 primary studies for the analysis from the umbrella review of 19 SRMAs [25]. The summary of the 100 primary studies is available in the Supplementary Material (Table S1). The main reason for exclusion of the 20 studies is that the study intervention did not meet the inclusion criteria. Fifty-five (55) unique resources were identified in the interventions, of which 18 were human resources and 37 were financial resources/ health spendings. These resources were grouped into the following sub-categories of medical, nursing, allied health, ancillary staff, and non-hospital staff for human resources: medical and non-medical food, general and non-standard equipment/services, media and education, and miscellaneous items/fees (Table 2).

**Figure 1:**
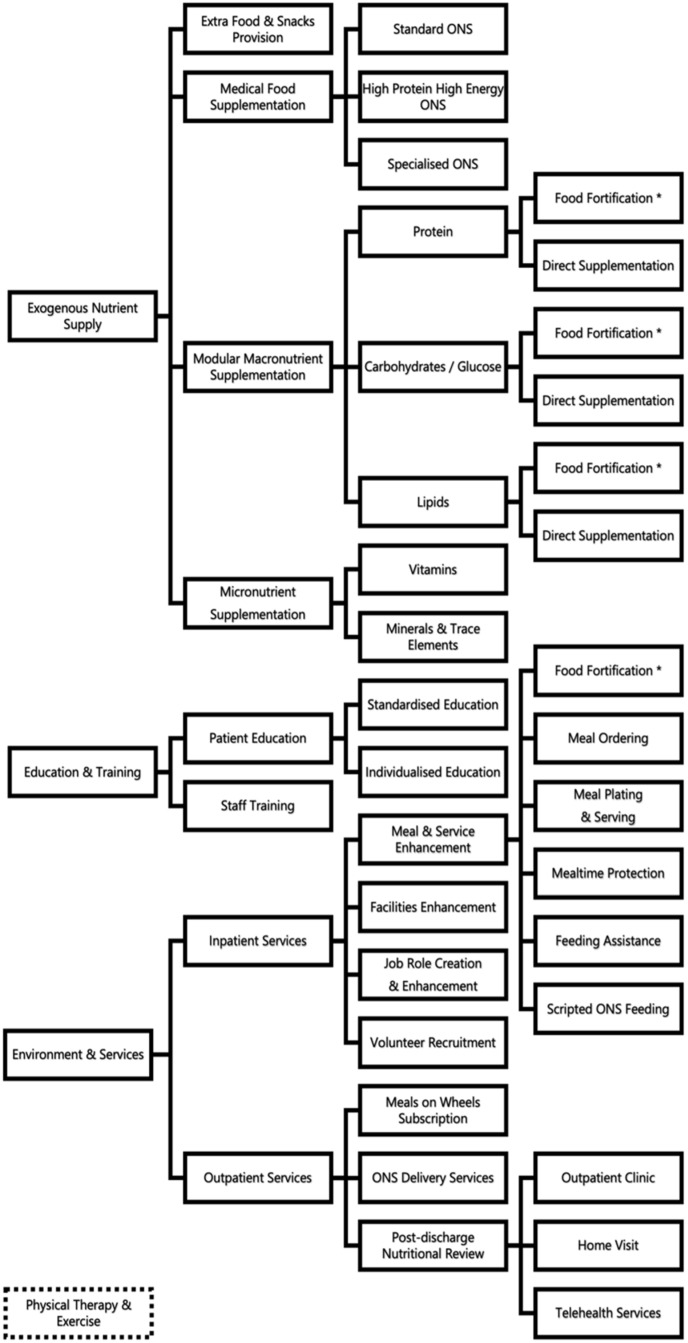
Nutritional strategies and target areas intervention in hospital settings. * Food fortification is used in the areas of (1) exogenous nutrient supply and (2) environment and services.

Fourteen (14) unique strategies were observed in the 100 primary studies to be implemented as a single intervention or in combination as multi-strategy interventions. These intervention strategies could be grouped broadly under the three main areas of education and training (ET), exogenous nutrient supply (EN), and environment and services (ES) (Figure 1).

The unique strategies identified include medical food supplementation, modular macronutrient supplementation through direct oral intake or food fortification, micronutrient supplementation through vitamins and/or minerals/trace elements, additional food or snacks provision, patient education and/or staff training, measurement of energy requirement with indirect calorimetry, meal service enhancement (through meal ordering, meal plating, food fortification, mealtime protection, and feeding assistance), new job role creation/enhancement, volunteer recruitment, MedPass/scripted feeding of oral nutritional supplements, facility enhancements of dining areas or wards, meals on wheels subscription, medical food delivery provision, post-discharge nutritional reviews in clinics or via home visits, and telehealth services.

Physical therapy and exercise were also commonly incorporated into interventions targeted at patients with malnutrition during hospitalization and post-discharge. Twelve (12) nutritional intervention studies have specific physical therapy or exercise interventions included, with the majority (90%) conducted by a physiotherapist or physical therapist, and the remaining by an exercise physiologist or sports trainer.

### 3.2. Strategies and Resources in Nutritional Interventions

Twenty (n = 20) studies included interventions that target all three areas of ET, ENS, and ES. Forty-six studies (n = 46) incorporated interventions targeting two areas [ET with ENS (n = 25), ET with ES (n = 15), and ENS with ES (n = 6)]. Thirty-four (n = 34) studies had interventions targeting only one specific area, with most studies targeting the provision of ENS via medical food (e.g., oral nutritional supplements) or general food (e.g., snacks at tea break or fortified food with main meals).

Interventions were mainly delivered in a hospital setting (n = 75) or hospital to community setting (n = 25). Most interventions delivered at least one of their components face-to-face in the hospital (n = 79), or in outpatient clinics and home visits on discharge (n = 23). Intervention strategies were also delivered via written materials (n = 46) and telephone contact (n = 18).

Amongst the 62 interventions targeting patients directly, 55% were individualized (n = 34) and the rest standardized (n = 28) nutritional interventions. For interventions targeted at staff or the environment (n = 38), only 14 studies (37%) provided details of training. The duration of interventions varied from three days to one year.

### 3.3. Complexity of Nutritional Interventions

Using the Medical Research Council 2021 Framework for complex interventions [24], 64 studies were classified as complex and the remaining 36 were simple interventions, with details presented in the Supplementary Data. Amongst the studies classified as complex interventions, 34 (53%) were found to be included in two or more SRMAs in the umbrella review [25].

### 3.4. Logistic Regression Model

Logistic regression analysis identified three variables that predict the complexity of an intervention, namely the numbers of (a) strategies used, (b) areas targeted by the nutritional intervention, and (c) healthcare professionals involved in carrying out the intervention. The odds of an intervention being classified as a simple intervention is 0.00138 with every 1 unit increase in the numbers of strategies used, the areas targeted by the nutritional intervention, and the healthcare professionals involved (Table 3). The multivariate regression model has a precision of 0.91 (sensitivity 0.95 and specificity of 0.83). Multicollinearity was not observed, with a VIF of approximately 1.0 for all three covariates.

Sensitivity analysis performed for interventions originating from RCTs only did not show any difference in the variables that predict the complexity of an intervention (Supplementary Material Table S2).

## 4. Discussion

This study aimed to systematically describe and analyze the resource utilization and components of nutritional interventions for adult malnutrition in hospital settings, and to determine the variables that predict the complexity of an intervention. We identified 14 unique strategies and 56 distinctive resources used in the 100 primary studies included in this study, demonstrating a wide range and substantial variation in what contributes to the complexity of interventions. The findings highlight the need for a more comprehensive approach to characterizing and evaluating nutritional interventions for future research trials and SRMAs investigating the effectiveness of interventions.

Our findings support the hypothesis that nutritional interventions can be systematically categorized into simple and complex based on their inherent characteristics. This categorization aids in understanding the resource implications and potential effectiveness of different interventions, underscoring the need for tailored approaches to nutritional therapy in hospitalized adults. The findings from this study can be used as a first screening step to differentiate between complex and simple nutritional interventions by researchers performing SRMAs or even narrative and scoping reviews, to allow for better categorization of interventions. The 14 strategies identified from the primary studies could be broadly grouped into the three major categories of ET, ENS, and ES. This finding supports the notion that a complex disease such as malnutrition requires a multifaceted approach in its prevention and treatment [1,2].

The majority of interventions included two or more intervention strategies, with ENS being the most commonly used strategy. This could be due to the relative ease of implementing and measuring the effects of nutrient supplementation, and the easy availability and access to medical food (ONS) and additional food/snacks. The sponsorship of such trials by the private industry could also explain the large number of such trials [28]. However, this may increase the potential for bias, as clinicians and researchers may associate any positive outcomes of interventions solely with the use of nutritional supplementation.

Nutritional interventions within the hospital settings tend to target various aspects of the cause of malnutrition, such as the social, financial, and nutrient intake aspects, and hence include more than one strategy, as shown by this conceptual paper. Furthermore, interactions between the various components of intervention or between the patients and intervention increase the complexity of the intervention [29].

Baldwin et al. [28] recently reported in an overview of SRMAs that the evidence for the effects of ONS in patients with or at risk of malnutrition is uncertain in SRMAs of trials using ONS as the main intervention. However, the majority of trials using ONS as the main intervention often do not report the adherence rate or how adherence to intervention is defined, as shown by our umbrella review [25]. Additionally, it is unknown whether improvements or no changes in outcomes observed are related to such highly heterogenous interventions that use multiple strategies. These limitations observed from the pooling of highly heterogenous studies have been persistently repeated, even in recent SRMAs [21,30,31].

One important implication of our findings is the need for a more transparent and systematic approach to the reporting and evaluation of nutritional interventions. The Medical Research Council 2021 Framework for complex interventions [24] provides a useful starting point for this endeavor, as it emphasizes the importance of considering the interacting components, context, and mechanisms of action of interventions [24]. By adopting this framework and the guidance on intervention complexity from the Cochrane handbook [32], researchers can better strengthen the design of SRMAs for nutritional interventions, leading to less heterogeneity in the pooled outcomes and more robust results that can be replicated.

The regression analysis demonstrated that the complexity of interventions is associated with the number of strategies used, areas targeted, and healthcare professionals involved. Surprisingly, individualization of intervention does not contribute to the complexity of the intervention. This could be due to factors such as consistency in approach, where individualization of intervention includes a consistent set of procedures, patient assessments, and decision-making processes [33]. Hence, although the nutritional care plan is individualized, the act of arriving at the care plan may not be complex. Furthermore, individualization only involves slight adjustments to a standard nutritional plan, such as switching a food item or another, or adding macro- or micronutrients to a patient’s usual diet [34]. While individualization of nutritional interventions did not significantly contribute to their complexity, it is crucial to consider the context and execution of the intervention. Highly trained professionals will likely streamline individualization processes, making them appear less complex, but still catering to the nuanced needs of patients. This underscores the need for a consistent, standardized approach in implementing individualized nutritional care [35].

Complex interventions may be more effective in addressing the multifactorial nature of malnutrition, as they can account for individual needs and preferences, as well as addressing multiple underlying causes [36]. However, complex interventions also require more resources, as shown by the results, and therefore, may be more challenging to implement, which could limit their feasibility and scalability in some settings [37,38]. Future research should determine the optimal balance between complexity of intervention and resource utilization, and explore methods to maximize the impact of interventions within resource constraints.

Additionally, we also highlighted the importance of examining the delivery methods and settings of nutritional interventions. While the majority of interventions were delivered in hospital settings, some involved community-based components or telehealth services post-discharge. This raises questions about the effectiveness of different delivery methods and settings, as well as the potential role of telehealth and other innovative approaches in improving the reach and impact of nutritional interventions. Digitalization and technology have been increasingly used in healthcare, including nutritional interventions. This has greatly facilitated individualization by using algorithms and databases to customize nutritional care plans of individuals [39,40], leading to a more efficient and less complex process. Further research is needed to address these questions and inform the design of future interventions.

This conceptual paper is not without limitations. Firstly, the analysis was based on studies selected from our recent umbrella review [25]. Although the umbrella review was performed in a systematic manner and included SRMAs and their relevant primary studies in the past 10 years, it was possible to overlook potential publications. Future research should consider a wider range of databases, registries, study protocols, and grey literature to encompass a broader spectrum of nutritional interventions. Our reliance on the Medical Research Council 2021 Framework [24] for classifying intervention complexity might not fully encapsulate the multifaceted nature of nutritional interventions. An expanded framework, incorporating nutritional-specific complexities, is recommended for future studies.

Lastly, the findings may be limited by the quality and reporting of the included studies, and therefore may not provide a complete picture of the resource utilization and components of nutritional interventions. As the umbrella review [25] focused primarily on RCTs for the meta-analysis, risk of bias was not performed for the observational or non-randomized clinical trials included in this conceptual paper. However, it is important to include these non-RCTs in the analysis for resource utilization and complexity analysis as there were a significant number of publications. To mitigate the possible effect from the use of non-RCTs in the regression analysis for complexity, we performed an additional sensitivity analysis with only RCTs and confirmed that there were no differences observed in the predictors for complexity.

## 5. Conclusions

This conceptual paper provides a novel and practical way of determining the complexity of nutritional intervention, as well as providing a classification method for the wide variety of interventions observed. The findings highlight the importance of considering the complexity of interventions, as well as the need for a more comprehensive approach to their development and evaluation for future studies and SRMAs. Further research is needed to assess the effectiveness of these complex interventions in various patient populations and settings, and to identify the optimal combination of resources and strategies for achieving the best clinical outcomes.

## Figures and Tables

**Table 1:** Characteristics and considerations to note of complex nutritional interventions.

	Criteria	Complex Nutritional Intervention
**1**	Number of interacting components within the experimental and control interventions To consider if there is any synergistic relationship or connectivity between the interacting components. To consider the presence of mediating and/or moderating factors that asserts an effect on the intervention.	Multiple interacting components between the intervention, environment, and individuals (the receiving or performing the intervention) involved. e.g., interaction will include educating subjects using newly updated educational materials or performing nutritional assessments via non-traditional forms of modality such as telehealth services. e.g., non-interaction will be handing out dietary pamphlets and informing the subjects to read through the information during their free time at home; or conducting a refresher course on nutrition screening to experienced nurses.
**2**	Number and difficulty of behaviours Number of components involved; the range of behaviours targeted by the intervention. Expertise and skills required by those delivering and receiving the intervention. Consider the possibility of behavior or actions of individuals or organizations affected by the intervention.	Expertise and skills required by those delivering and training or reinforcement by those receiving the intervention. Individualized or tailored interventions provided by experienced individual/staff who have training or expertise in the delivery are not considered as a difficulty for this criterion. e.g., individualized dietary counseling that is routinely performed by clinical dietitians, physical therapy by physiotherapists, and measurement of resting energy expenditure by trained nurses in indirect calorimetry. Premorbid characteristics of subjects and environment where the intervention is taking place may affect the delivery of the intervention and will be considered as a difficulty. e.g., education level and socioeconomic status of subjects taking part in an intervention that requires the use of videoconferencing.
**3**	Number of groups or organizational levels The number of groups, settings, or levels targeted by the intervention. To consider if the effects of the intervention appear to be context-dependent, i.e., the conditions needed to realize its mechanisms of change and/or the resources required to support intervention reach and impact in real-world implementation.	A complex nutritional intervention intervenes and disrupts the functioning of complex systems by changing relationships, modifying or transforming established practices, and redistributing resource allocations at hospital or patient level. This may include establishing protected meal timing in hospitals, which may lead to disruption of mealtime procedures for other departments or require policy changes to bring about the intervention. Additionally, the effects of intervention may be context-dependent. e.g., policy timing, leadership, work culture, resource allocation, and interpersonal relationships within the organization.
**4**	The number and variability of outcomes To consider any non-linear relationships of input (intervention) and output (outcomes) and if the components of intervention may bring more benefit, neutralization, or reduction in effect (outcomes) when performed in combination compared to in isolation.	In general, complex nutritional interventions have multiple components designed to affect multiple outcomes such as improving quality of life, and reducing infections and mortality in malnourished patients. These outcomes may differ between patient populations, such as between those in critical care and general medical populations. The intervention may have other effects that are unanticipated. ii. e.g., increased hospitalization costs while reducing mortality and complications due to costs of a complex intervention, or reduced readmissions and therefore healthcare costs. Note: for the purpose of this paper, we did not consider the number and variability of outcomes as necessary factors for a complex intervention as outcome data were not collected for this analysis.
**5**	The degree of flexibility or individualization of the intervention allowed. To consider if there are any feedback loops where any change may modify (e.g., reinforce, promote, maintain, or reduce) any aspects of the intervention.	Any intervention that targets the individual patient and has the flexibility to be tailored based on the individual patient’s needs.Feedback from patients will translate into the change in intervention, e.g., patients unable to tolerate sufficient volume of nutritional supplements will receive a higher energy density supplement. Excludes standardized intervention that is provided for all patients e.g., a standard dietary education pamphlet, and a fixed volume of ONS supplied daily that is not affected by a feedback loop.

**Table 2:** Resources utilized in nutritional interventions.

Human	Financial	
**Allied Health Professionals** Dietitian/Nutritionist Physiotherapist Occupational Therapist Speech Therapist Exercise Physiologist Sports Trainer Pharmacist **Nursing Staff** Specialty Nurses/Case Managers Registered Nurse Assistant Nurse **Medical Staff** Hospital Specialist General Practitioner **Ancillary Staff** Healthcare Attendant Nursing Aide Food Service Staff Therapist Assistant Dietetic Assistant **Others** Volunteer	**Medical Food** Oral Nutritional Supplements (Standard, High Protein High Energy, Specialized) Modular Macronutrients (Protein, Carbohydrates, Lipid) Micronutrients (Vitamins, Minerals, Trace Elements) **Non-Medical Food** Additional Hospital Meals Food Fortification Ingredients (e.g., Plant Oils, Dextrose, Whey Protein) Home-made Supplements (e.g., drinks and puddings based on milk and eggs) Commercial protein and energy drinks Commercial Desserts and Snacks (e.g., Cookies, Cake, Ice-cream, puddings, High Protein yoghurt, High Protein Bread, Crackers and Cheese) Drinks (e.g., Cocoa with whipped cream, Flavored milk) **Media and Education** Advertising Posters Electronic Media Patient Brochures Nutrition Education Sheets for Staff/Learning Packs for Volunteer Multidisciplinary In-services Procedural Documentations Educational Learning Packs for Staff Training **Miscellaneous Items/Fees** Transport Claims for Home Visits by Staff Uniforms for Volunteers Waiver of Car Park Charges for Volunteers Certificates for Volunteer Renovation Fees for Major Upgrades to Dining Area Exercise Equipment for Patients (Free Weights, Resistance Bands)	**General Equipment, Technology and Services** 1. Weighing Scales 2. Stadiometer 3. Knee-length Caliper 4. Measuring Tape 5. Standard Laboratory Tests (e.g., blood tests for common nutritional markers) 6. Delivery Fees for Medical Food and Meals on Wheels 7. Glass Door Refrigerator for Snacks and Beverages 8. Purple Lid and Red Trays to Alert Nursing Staff and Volunteers for Feeding Assistance. 9. Ward Door Signages for Mealtimes 10. Tablecloths and small vases for decoration 11. Colored Tray Mats and Napkins for Dining Hall **Non-Standard Equipment, Technology, and Services** Skinfold Calipers/Mechanical Pinch Gauge Hydraulic Hand Dynamometer Specialized Laboratory Tests (e.g., Muscle Biopsy) Specialized Medical Equipment (e.g., DEXA scans, Computed Tomography Scans, Bio-impedance Analysis) Software for Dietary Assessment (e.g., FoodWorks (FoodWorks Edition 10), DietPlan (DietPlan 6.0)) Upgrades of Meal Ordering System (Hardware and Software)

**Table 3:** Complex versus simple interventions. Logistic regression model using identified variables.

Coefficients										
						Wald Test			95% CI (OR Scale)	
Model	Parameter	Estimate	Standard Error	Odds Ratio (Exp β)	z	Wald Statistic	Degrees of Freedom	*p*	Lower Bound	Upper Bound
	(Intercept)	−6.58	1.47	1.38 × 10^−3^	−4.49	20.13	1	<0.001	0	0.02
Number of Healthcare Professionals Involved		1.16	0.33	3.2	3.55	12.63	1	<0.001	1.68	6.07
Number of Intervention Areas Targeted		1.75	0.53	5.74	3.27	10.72	1	0.001	2.02	16.32
Number of Interventions Employed		0.82	0.3	2.26	2.69	7.25	1	0.007	1.25	4.1
Note. Complex intervention coded as class 1.										
**Multicollinearity Diagnostics**										
		Tolerance	VIF							
Number of Healthcare Professionals Involved		0.91	1.1							
Number of Intervention Areas Targeted		0.99	1.01							
Number of Interventions Employed		0.9	1.11							
**Performance Metrics**										
		Value								
Accuracy		0.91								
AUC		0.95								
Sensitivity		0.95								
Specificity		0.83								
Precision		0.91								

## Data Availability

The data that support the findings of this study are available in the Supplementary Material of this article.

## Supplementary Materials

The following supporting information can be downloaded at: https://www.mdpi.com/article/10.3390/healthcare12070765/s1, Table S1: Studies included in the analysis; Table S2: Sensitivity Analysis for Complex versus Simple Interventions. Logistic regression model using identified variables.
