# Supplementary material for: A Conceptual Study on Characterizing the Complexity of Nutritional Interventions for Malnourished Older Adults in Hospital Settings: An Umbrella Review Approach
Source: Healthcare (Basel). 2024 Mar 31;12(7):765. doi: 10.3390/healthcare12070765 (PMC11011329; doi:10.3390/healthcare12070765)
Supplement: Supplementary file 1 [file healthcare-12-00765-s001.zip › healthcare-2862451-supplementary.pdf]

Supplementary Materials

Table S1. Studies included in the analysis

Study	Year	Study Type (RCT =1, NRSI = 2, cRCT = 3)	Control	Intervention	Complexity of Intervention (Simple = 1, Complex = 2)
Abalan F, Manciet G, Dartigues JF, Decamps A, Zapata E, Saumtally B, et al. Nutrition and SDAT. *Biological Psychiatry* 1992;31(1):103-5.	1992	1	1. Standard hospital diet	1. Standard hospital diet<br>2. Oral nutrients (multivitamin, magnesium, calcium, phosphorus, L-tryptophan, Trace elements, B1/6/12, Folic Acid)<br>3. ONS x 2 serves/d (400kcal/d and protein 22.5g/d)	1
Anbar R, Beloosesky Y, Cohen J, Madar Z, Weiss A, Theilla M, et al. Tight calorie control in geriatric patients following hip fracture decreases complications: a randomized, controlled study. *Clinical Nutrition* 2014;33(1):23-8.	2014	1	1. Standard care	1. Provision of energy with a goal determined by repeated REE measurements using indirect calorimetry<br>2. Additional ONS if energy not met (~220kcal/d extra)	2
Arias S, Bruzzone I, Blanco V, Inchausti M, Garcia F, Casavieja G, et al. Identification and early nutritional support in hospitalized malnourished patients. *Nutricion Hospitalaria* 2008;23(4):348-53.	2008	1	1. Standard hospital diet	1. Standard hospital diet and ONS	1
Banerjee AK, Brocklehurst JC, Wainwright H, Swindell R. Nutritional status of long-stay geriatric in-patients: effects of a food supplement (Complan). *Age and Ageing* 1978;7(4):237-43.	1978	1	1. Dietary education	1. ONS (265kcal/18.6g protein)<br>2. Dietitian spent time (5 days) in the ward looking after patients	1

Study	Year	Study Type (RCT =1, NRSI = 2, cRCT = 3)	Control	Intervention	Complexity of Intervention (Simple = 1, Complex = 2)
Barton AD, Beigg CL, Macdonald IA, Allison SP. A recipe for improving food intakes in elderly hospitalized patients. *Clinical Nutrition* 2000;19(6):451-4.	2000	2	NA	1. Energy-enriched menu (addition of 200 kcal/d and protein 5 g/d)<br>2. Addition of a cooked breakfast (200 kcal/d and protein 12 g/d per day)	1
Beck A, Andersen UT, Leedo E, Jensen LL, Martins K, Quvang M, et al. Does adding a dietician to the liaison team after discharge of geriatric patients improve nutritional outcome: a randomised controlled trial. *Clin Rehabil* 2015 Nov;29(11):1117-28.	2015	1	1. Discharge Liaison-Team visit without dietitian	1. Pre-discharge, dietitians evaluated the need for oral supplements and prescribed them as necessary.<br>2. Post-discharge, individualized assessments were conducted, focusing on diet, activity, and weight to inform a personalized nutrition care plan, which could involve coordinating with meal providers for high-energy, protein-rich meals.<br>3. A total of three home visits were scheduled to explore options for supplements and micronutrients, such as vitamin D and calcium, partially covered by health insurance, to optimize nutritional intake.	2
Beck AM, Kjær S, Hansen BS, Storm RL, Thal-Jantzen K, Bitz C. Follow-up home visits with registered dietitians have a positive effect on the functional and nutritional status of geriatric medical patients after discharge: a randomized controlled trial. *Clinical Rehabilitation* 2012; 27(6):483–93.	2012	1	1. Three follow-up visits by general practitioners alone.	1. Patients received three home visits for individualized nutritional counseling by a registered dietitian, complemented by three follow-up visits from general practitioners.<br>2. Strategies to optimize nutritional status included supplementation with energy- and protein-dense meals, ONS, and micronutrient recommendations such as vitamin D and calcium.	2

Study	Year	Study Type (RCT =1, NRSI = 2, cRCT = 3)	Control	Intervention	Complexity of Intervention (Simple = 1, Complex = 2)
Beelen J, Vasse E, Janssen N, Janse A, de Roos NM, de Groot LCPGM. Protein-enriched familiar foods and drinks improve protein intake of hospitalized older patients: A randomized controlled trial. *Clin Nutr.* 2018 Aug;37(4):1186-1192	2018	1	1. Standard energy and protein rich hospital menu	1. Received various protein-enriched products replacing regular products or added to the menu	2
Bell JJ, Bauer JD, Capra S, Pulle RC. Multidisciplinary, multimodal nutritional care in acute hip fracture inpatients – results of a pragmatic intervention. *Clin Nutr.* 2014;33(6):1101-1107.	2014	2	NA	1. Nutrition assistants implemented a twice-weekly review protocol with scripted high-calorie supplements and selective mid-meals.<br>2. Champions led a multimedia strategy, including posters and electronic media, to boost nutritional awareness.<br>3. The strategy also featured in-services and documentation to educate patients, carers, and the multidisciplinary team.	2
Bonilla-Palomas JL, et al. Nutritional intervention in malnourished hospitalized patients with heart failure. *Arch Med Res* 2016;47(7):535-40.	2016	1	1. Standard care	1. A 6-month nutritional intervention was led by a physician specialist and a nutritionist, starting at admission.<br>2. The protocol focused on diet optimization, specific recommendations, and supplement prescriptions when goals were unmet.<br>3. Supplement choices were tailored to patient requirements and co-morbidities.	2
Botella-Carretero JI, Iglesias B, Balsa JA, Zamarron I, Arrieta F, Vazquez C. Effects of oral nutritional supplements in	2008	1	1. Standard diet	1. Intervention 1: Standard diet + extra protein 36g/d<br>2. Intervention 2: Standard diet + extra 500kcal/d + + extra protein 36g/d	1

Study	Year	Study Type (RCT =1, NRSI = 2, cRCT = 3)	Control	Intervention	Complexity of Intervention (Simple = 1, Complex = 2)
normally nourished or mildly undernourished geriatric patients after surgery for hip fracture: a randomized clinical trial. *Journal of Parenteral and Enteral Nutrition* 2008;32(2):120-8.					
Broqvist M, et al. Nutritional assessment and muscle energy metabolism in severe chronic congestive heart failure–effects of long-term dietary supplementation. *Eur Heart J* 1994;15(12):1641-50.	1994	1	1. Placebo ONS	1. ONS (500 ml/d, protein 30 g/d, 750 kcal/d)	1
Brown, Helen; Jones, Lucy (2009). The role of dining companions in supporting nursing care. *Nursing Standard*, 23(41), 40–46. doi:10.7748/ns2009.06.23.41.40.c7049	2009	2	NA	1. Six nursing student volunteers were locally recruited and trained via an interactive workshop led by a speech therapist and dietitian.<br>2. Speech therapists liaised with nurses for initial two weeks to offer volunteer support and guidance.<br>3. Ward nurses handled nutritional care planning and delegated patient feeding.	2
Buys DR, Flood KL, Real K, Chang M, Locher JL. Mealtime assistance for hospitalized older adults - a report on the spoons volunteer program. *J Gerontol Nurs* 2013;39(9):18- 22.	2013	2	NA	1. Volunteers were trained in three sessions by a unit coordinator and occupational therapist (OT).<br>2. On average, volunteers spent 47.8 minutes per encounter, resulting in cost savings.<br>3. Savings averaged USD 11.94 per encounter compared to a patient care technician (PCT) and USD 26.00 compared to a registered nurse (RN).	2

Study	Year	Study Type (RCT =1, NRSI = 2, cRCT = 3)	Control	Intervention	Complexity of Intervention (Simple = 1, Complex = 2)
Campbell KL, Webb L, Vivanti A et al. (2013) Comparison of three interventions in the treatment of malnutrition in hospitalised older adults: a clinical trial. *Nutr Diet* 70, 325–331.	2013	2	NA	1. Diet Assistants (DAs) at AUD 28.10/hr facilitated traditional and mid-meal services, offering up to four energy-enriched snacks per day.<br>2. Snacks provided 70-120 kcal and 0-6 g protein, sourced from a mid-meal trolley of high-protein and/or high-energy options.<br>3. Selections included various items like flavored milk, chocolate biscuits, and nuts, delivered between main meals.	1
Cano-Torres EA, et al. Impact of nutritional intervention on length of hospital stay and mortality among hospitalized patients with malnutrition: a clinical randomized controlled trial. *J Am Coll Nutr* 2017;36(4):235-9	2017	1	1. Standard nutritional management according to the Hospital Nutrition Department.	1. Individualized nutrition plan according to energy and protein (1.0–1.5 g/kg) intake requirements<br>2. Dietary advice based on face-to-face interviews with patients and their caregivers or family members.	2
Casals C, García-Agua-Soler N, Vázquez-Sánchez M, Requena-Toro MV, Padilla-Romero L, Casals-Sánchez JL. Randomized Clinical Trial of Nutritional Counselling for Malnourished Hospital Patients. *Rev Clin Esp (Barc)*. 2015;215(6):308-14.	2015	1	1. Standard care	1. Dietary counseling x 3 by case manager nurses during the hospital stay and upon discharge (repeated at first month and subsequently every two months) (n=52)	2

Study	Year	Study Type (RCT =1, NRSI = 2, cRCT = 3)	Control	Intervention	Complexity of Intervention (Simple = 1, Complex = 2)
Chen CC-H, Yang Y-T, Lai I-R, Lin B-R, Yang C-Y, Huang J, et al. Three nurse administered protocols reduce nutritional decline and frailty in older gastrointestinal surgery patients: a cluster randomized trial. *J Am Med Dir Assoc* 2019;20(5):524-9.e3.	2019	3	1. Standard hospital care provided by physicians and nurses on the general surgery wards.	1. Hospital-based mHELP comprising three nursing protocols: early mobilization, oral and nutritional assistance, and orienting communication.<br>2. No ONS	2
de Sousa OL, Amaral TF. Three-week nutritional supplementation effect on long-term nutritional status of patients with mild Alzheimer disease. *Alzheimer Disease and Associated Disorders* 2012;26(2):119-23. [PMID: 21878804]	2012	1	1. Standard dietetic advice	1. ONS x 1 serve/d<br>2. Standard dietetic advice	1
Delmi M, Rapin CH, Bengoa JM, Delmas PD, Vasey H, Bonjour JP. Dietary supplementation in elderly patients with fractured neck of the femur. *Lancet* 1990;335(8696):1013-6.	1990	1	1. Standard diet	1. ONS x 1 serve/d<br>2. Standard hospital diet	1
Dennis M, Lewis S, Cranswick G, Forbes J. FOOD: a multicentre randomised trial evaluating feeding policies in patients admitted to hospital with a recent stroke. *Health Technology Assessment* 2006;10:iii-iv, ix-x, 1-120. Dennis MS, Lewis SC, Warlow C. Routine oral nutritional supplementation for stroke patients in hospital (FOOD): a	2005	1	1. Standard diet	1. Three doses of 120 ml 1.5 kcal/ml ONS given daily and prescribed on the drug chart and normal hospital diet until hospital discharge. ONS decanted into a cup for patient<br>2. A wide variety of ONS of different consistencies was sourced for patients	1

Study	Year	Study Type (RCT =1, NRSI = 2, cRCT = 3)	Control	Intervention	Complexity of Intervention (Simple = 1, Complex = 2)
multicentre randomised controlled trial. *Lancet* 2005; Vol. 365, issue 9461:755-63.					
Deutz NE, et al. Readmission and mortality in malnourished, older, hospitalized adults treated with a specialized ONS: a randomized clinical trial. *Clin Nutr* 2016;35(1):18-26.	2016	1	1. Standard-of-care plus placebo supplement	1. Standard-of-care plus ONS with high protein and HMB	1
Dube, L., Paquet, C., Ma, Z., Kergoat, M., Ferland, G., 2007. Nutritional implications of patient-provider interactions in hospital settings: evidence from a within subject assessment of mealtime exchanges and food intake in elderly patients. *Eur. J. Clin. Nutr.* 61, 664–672.	2007	2	NA	1. Communal dining room<br>2. All participants were assigned to one of six tables, where they ate all their meals in the company of up to three fellow patients<br>3. A team of care providers (nurses and orderlies) was present to perform meal service and provide meal assistance	1
Duncan, D.G., Beck, S.J., Hood, K., Johansen, A., 2006. Using DAs to improve the outcome of hip fracture: a randomised controlled trial of nutritional support in an acute trauma ward. *Age Ageing* 35, 148–153.	2006	1	1. Conventional pattern of nurse- and dietitian-led care	1. Dietetic assistant	1

Study	Year	Study Type (RCT =1, NRSI = 2, cRCT = 3)	Control	Intervention	Complexity of Intervention (Simple = 1, Complex = 2)
Ekinci, O., Yanik, S., Terzioğlu Bebitoğlu, B., Yilmaz Akyüz, E., Dokuyucu, A., & Erdem, Ş. (2016). Effect of Calcium β-Hydroxy-β-Methylbutyrate (CaHMB), vitamin D, and protein supplementation on postoperative immobilization in malnourished older adult patients with hip fracture: A randomized controlled study. *Nutrition in Clinical Practice*, 31, 829–835. https ://doi.org/10.1177/0884533616629628	2016	1	1. Standard postoperative nutrition	1. ONS (high protein + HMB) x 2 serves/d in addition to standard postoperative nutrition	1
Espaulella J, Guyer H, Diaz-Escriu F, Mellado-Navas JA, Castells M, Pladevall M. Nutritional supplementation of elderly hip fracture patients. A randomized, double-blind, placebo controlled trial. *Age and Ageing* 2000;29(5):425-31.	2000	1	1. Placebo ONS (152kcal 0g protein) x 1 serve/d	1. ONS (149 kcal/d 20g/d protein Ca Vit D3) x 1 serve/d	1
Faxen-Irving G, Cederholm T. Energy dense oleic acid rich formula to newly admitted geriatric patients--feasibility and effects on energy intake. *Clinical Nutrition* 2011; 30(2):202-8.	2011	1	1. Standard care	1. 30 ml fat emulsion (Calogen) x 3 serves/d (420 kcal/d)	1

Study	Year	Study Type (RCT =1, NRSI = 2, cRCT = 3)	Control	Intervention	Complexity of Intervention (Simple = 1, Complex = 2)
Feldblum I, et al. Individualized nutritional intervention during and after hospitalization: the nutrition intervention study clinical trial. *J Am Geriatr Soc* 2011;59(1):10-7.	2011	1	1. One group had a single hospital meeting with a dietitian and received advised supplementation during hospitalization.<br>2. A second group received standard care with unreported supplement intake.<br>3. Both groups were later combined to form a single control group for the study.	1. Patients received one inpatient and two home dietary counseling sessions by a dietitian, with follow-up assessments at 3 and 6 months post-discharge by a coordinator.<br>2. Coordinator also made six phone calls for monitoring, and patients were prescribed 1-2 servings of 1.5 kcal/ml supplements up to 6 months, including options like liquid or pudding.<br>3. When micronutrient intake was below 75% of the Daily Recommended Intake (DRI), appropriate vitamin and mineral supplements were provided, with the amount being unspecified.	2
Gariballa S, et al. A randomized, double-blind, placebo-controlled trial of nutritional supplementation during acute illness. *Am J Med* 2006;119(8): 693-9.	2006	1	1. Normal hospital diet plus a placebo daily for 6 weeks.	1. Normal hospital diet plus ONS 400ml/d for 6 weeks	1
Gariballa SE, Parker SG, Taub N, Castleden CM. A randomized, controlled, a single-blind trial of nutritional supplementation after acute stroke. *Journal of Parenteral and Enteral Nutrition* 1998;22(5):315-9.	1998	1	1. Standard care	1. Hospital diet plus a twice daily ONS of >400 mL (600 kcal/d and protein 20 g/d) at 3:00 PM and 8:00 PM each day for 4 weeks or until death or discharge.	1
Gazzotti C, et al. Prevention of malnutrition in older people during and after hospitalisation: results from a randomised controlled clinical trial. *Age Ageing* 2003;32(3):321-5.	2003	1	1. Standard care	1. 200 ml sweet or salty ONS twice daily (500 kcal/d and protein 21g/d) throughout hospitalization and convalescence.	1

Study	Year	Study Type (RCT =1, NRSI = 2, cRCT = 3)	Control	Intervention	Complexity of Intervention (Simple = 1, Complex = 2)
Hayward J. Ward nutrition coordinators to improve patient nutrition in hospital. *Br J Nurs*. 2003;12(18):1081-1089.	2003	2	NA	1. Each ward had a full-time nutrition coordinator who underwent a one-week multidisciplinary induction program, including training with dietitians, caterers, speech and occupational therapists, and a nutrition nurse specialist.<br>2. The program encompassed various aspects such as therapeutic diets, feeding aids, patient assessment, and documentation, featuring hands-on activities like tasting sessions and swallowing assessments.<br>3. Ongoing support for the coordinators was agreed to be provided by both the clinical team and the nutrition nurse specialist.	2
Hickson M, Bulpitt C, Nunes M, Peters R, Cooke J, Nicholl C, et al. Does additional feeding support provided by health care assistants improve nutritional status and outcome in acutely ill older in-patients? a randomised control trial. *Clin Nutr* 2004;23(1):69-77	2004	1	1. Standard care	1. Patients received additional nutritional care from a trained healthcare assistant (HCA) upon randomization.<br>2. All patients were prescribed medical and nutritional therapy as standard care.<br>3. The only variable was the level of feeding support provided by the trained HCA.	1
Hickson, M., Connolly, A., Whelan, K., 2011. Impact of protected mealtimes on ward mealtime environment, patient experience and nutrient intake in hospitalised patients. *J. Hum. Nutr. Diet.* 24, 370–374. doi:http://dx.doi.org/10.1111/j.1365-277X.2011.01167.x.	2011	2	NA	1. Protected mealtimes were implemented via a guideline document and disseminated through the nursing directorate.<br>2. Notices were posted on the intranet to inform staff and stakeholders.<br>3. Large signs were displayed to indicate when protected mealtimes were in progress.	2

Study	Year	Study Type (RCT =1, NRSI = 2, cRCT = 3)	Control	Intervention	Complexity of Intervention (Simple = 1, Complex = 2)
Hoekstra JC, Goosen JH, de Wolf GS, Verheyen CC. Effectiveness of multidisciplinary nutritional care on nutritional intake, nutritional status and quality of life in patients with hip fractures: a controlled prospective cohort study. *Clin Nutr* 2011 Aug;30(4):455-61.	2011	1	1. Standard nutritional care	1. Multidisciplinary nutritional care that focused on nutritional support during hospitalization and a transfer of nutritional care after discharge.	2
Hogarth MB, et al. Nutritional supplementation in elderly medical in-patients: a double-blind placebo-controlled trial. *Age Ageing* 1996;25(6):453-7.	1996	1	1. Placebo energy and active vitamin supplementation<br>2. Placebo energy and placebo vitamin supplementation.	1. Active energy and active vitamin supplementation<br>**2. Active energy and placebo vitamin supplementation**	1
Holst M, Beermann T, Mortensen MN, Skadhauge LB, Køhler M, Lindorff-Larsen K, et al. Optimizing protein and energy intake in hospitals by improving individualized meal serving, hosting and the eating environment. *Nutrition* 2017;34:14-20.	2017	2	NA	1. Healthcare professionals (HCPs) including nursing staff and kitchen hostesses received training as part of the intervention.<br>2. The training, conducted by the nutrition support team and hospital nutrition board, covered topics such as aesthetic meal serving, portion sizes, and patient assistance during meals.<br>3. Special emphasis was placed on catering to individual preferences and offering targeted meal assistance.	2

Study	Year	Study Type (RCT =1, NRSI = 2, cRCT = 3)	Control	Intervention	Complexity of Intervention (Simple = 1, Complex = 2)
Holyday M, et al. Malnutrition screening and early nutrition intervention in hospitalised patients in acute aged care: a randomised controlled trial. *J Nutr Health Aging* 2012;16(6):562-8.	2012	1	1. Usual nutrition care provided.<br>2. Control group only seen by the clinical dietitian if and when referred by medical or other health professionals, and if referred, the same care plan was implemented as for the intervention patients.	1. The Malnutrition Care Plan (MCP) included texture-modified and fortified hospital meals, prescription of nutrient-dense supplements, and meal assistance flagging by ward staff.<br>2. Education on optimizing nutrition was provided to patients and carers, and referrals were made for discharge planning.<br>3. The MCP was customized according to individual patient needs, based on the clinical dietitian's assessment and prescription.	2
Houwing RH, Rozendaal M, Wouters-Wesseling W, Beulens JW, Buskens E, Haalboom JR. A randomised, double-blind assessment of the effect of nutritional supplementation on the prevention of pressure ulcers in hip-fracture patients. *Clinical Nutrition* 2003;22(4):401-5.	2003	1	1. Placebo ONS (non-caloric, water-based drink containing only sweeteners, colorants, and flavorings)	1. 400ml high-protein ONS enriched with arginine, zinc, and antioxidants starting immediately postoperatively for a period of 4 weeks or until discharge.	1
Howson FFA, Robinson SM, Lin SX, Orlando R, Cooper C, Sayer AAP, et al. Can trained volunteers improve the mealtime care of older hospital patients? An implementation study in one English hospital. *BMJ Open* 2018;8(8).	2018	2	NA	1. After competency assessment, volunteers were met by the research team during their next two mealtime shifts to address any concerns and ensure acclimation to the ward.<br>2. Semi-annual "coffee and cake" meetings were held to discuss implementation progress and address volunteer concerns; volunteers typically assisted 2-3 patients per session, including feeding one.	2

Study	Year	Study Type (RCT =1, NRSI = 2, cRCT = 3)	Control	Intervention	Complexity of Intervention (Simple = 1, Complex = 2)
				3. Implementation costs over the 15-month study included 13 training sessions, individual competency sessions, and one hour of administrative support per volunteer for coordination.	
Huang CS, Dutkowski K, Fuller A, Walton K. Evaluation of a pilot volunteer feeding assistance program: influences on the dietary intakes of elderly hospitalised patients and lessons learnt. *J Nutr Health Aging* 2015;19(2): 206-10.	2015	2	NA	1. Volunteers trained by dietetics, speech pathology and nursing staff	2
Huxtable, S., Palmer, M., 2013. The efficacy of protected mealtimes in reducing mealtime interruptions and improving mealtime assistance in adult inpatients in an Australian Hospital. *Eur. J. Clin. Nutr.* 67, 904–910.	2013	2	NA	1. Staff training occurred approximately 5-7 months before and 5-6 months after the intervention, with volunteers aiding mealtime on four wards post-implementation. The nursing unit manager acted as an informal champion.<br>2. Four in-service educational sessions were conducted between 1-13 months post-protected mealtimes program (PMP) implementation, focusing on effective meal tray delivery and patient assistance.<br>3. Speech pathologists restructured clinics to allocate mealtime appointments for specific patient groups, and volunteers were deployed up to 8 months post-PMP to assist with meals.	2

Study	Year	Study Type (RCT =1, NRSI = 2, cRCT = 3)	Control	Intervention	Complexity of Intervention (Simple = 1, Complex = 2)
Huynh DT, et al. Effects of oral nutritional supplementation in the management of malnutrition in hospital and post-hospital discharged patients in India: a randomised, open-label, controlled trial. *J Hum Nutr Diet* 2015;28(4): 331-43.	2015	1	1. Dietary education	1. Prescription of nutritional supplements and dietary education were administered; 100% of participants received the supplements.<br>2. Dietary counseling was provided by a dietitian.<br>3. Three sessions of dietary counseling were conducted at baseline, week 4, and week 8 of the intervention.	2
Johansen N, Kondrup J, Plum LM, Bak L, Norregaard P, Bunch E, et al. Effect of nutritional support on clinical outcome in patients at nutritional risk. *Clinical Nutrition* 2004;23(4):539-50.	2004	1	1. Received nutritional care according to the usual procedure of the department.	1. The team provided daily attention to motivate both patients and staff for nutritional adherence.<br>2. Tailoring of the nutritional plan involved estimating protein and energy requirements, and coordinating food orders with patients.<br>3. The team also provided guidance on transitioning to alternative feeding methods such as tube feeding or parenteral nutrition when necessary.	2
Keele AM, Bray MJ, Emery PW, Duncan HD, Silk DBA. Two phase randomised controlled clinical trial of postoperative oral dietary supplements in surgical patients. *Gut* 1997;40(3):393-9.	1997	1	1. Standard hospital diet only	1. Standard hospital diet supplemented ad libitum with ONS (Fortisip, Nutricia, Holland), both in- and outpatient	1
Larsson J, Unosson M, Ek AC, Nilsson L, Thorslund S, Bjurulf P. Effect of dietary supplement on nutritional status and clinical outcome in 501 geriatric patients--a randomised study. *Clin Nutr.* 1990	1990	1	1. Standard hospital diet	1. ONS (400 kcal/d) as well as a standard hospital diet	1

Study	Year	Study Type (RCT =1, NRSI = 2, cRCT = 3)	Control	Intervention	Complexity of Intervention (Simple = 1, Complex = 2)
Aug;9(4):179-84. doi: 10.1016/0261-5614(90)90017-m. PMID: 16837353.					
Lassen KO, Grinderslev E, Nyholm R. Effect of changed organisation of nutritional care of Danish medical inpatients. *BMC Health Serv Res.* 2008;8(1):1-13.	2008	2	NA	1. A specialist role for nutritional healthcare assistants was established to ensure the nutritional and fluid needs of at-risk patients were met post-assessment.<br>2. Training involved a one-month program aimed at upskilling social and healthcare assistants to the level of nutritional and healthcare assistants.<br>3. The focus of the role and training was on providing targeted nutrition care to patients identified as nutritionally at-risk	2
Lassen KO, Kruse F, Bjerrum M, Jensen L, Hermansen K. Nutritional care of Danish medical inpatients: effect on dietary intake and the occupational groups' perspectives of intervention. *Nutr J* 2004;3(1):12.	2004	2	NA	1. The kitchen introduced new diet regimens during the study period as an additional variable.<br>2. Nurses received four training meetings to acquaint them with new forms and usage protocols; the timing of these meetings was not specified.<br>3. Weekly support was provided by investigators to the nursing staff, though the frequency and duration were not detailed.	2

Study	Year	Study Type (RCT =1, NRSI = 2, cRCT = 3)	Control	Intervention	Complexity of Intervention (Simple = 1, Complex = 2)
MacFie J, Woodcock NP, Palmer MD, Walker A, Townsend S, Mitchell CJ. Oral dietary supplements in pre- and postoperative surgical patients: a prospective and randomized clinical trial. *Nutrition* 2000;16(9):723-8.	2000	1	1. Group IV did not receive any supplements.	1. Group I received oral dietary supplements (ODSs) both pre- and postoperatively in addition to their normal diet.<br>2. Group II was administered ODSs exclusively in the preoperative period.<br>3. Group III received ODSs only during the postoperative period.	2
Malafarina V, Uriz-Otano F, Malafarina C, Martinez JA, Zulet MA. Effectiveness of nutrition supplementation on sarcopenia and recovery in hip fracture patients. A multi-centre randomized trial. *Maturitas*. 2017 Jul;101:42-50. doi: 10.1016/j.maturitas.2017.	2017	1	1. Standard diet + rehabilitative therapy	1. Standard diet plus ONS x 2 serves/d (with HMB) + rehabilitative therapy	2
Manning F, Harris K, Duncan R, Walton K, Bracks J, Larby L, et al. Additional feeding assistance improves the energy and protein intakes of hospitalised elderly patients. A health services evaluation. *Appetite* 2012;59(2):471-7.	2012	2	NA	1. Nurses spent a mean time of 6 min with patients during mealtimes providing assistance and only 4.7 min at lunchtime. Volunteers provided more than double this amount of time	1
Maunder K, Lazarus C, Walton K, Williams P, Ferguson M, Beck E. Energy and protein intake increases with an electronic bedside spoken meal ordering system compared to a paper menu in hospital patients. *Clin Nutr ESPEN*. 2015;10(4):e134-e139.	2015	2	1. Paper menu	1. Electronic bedside spoken meal ordering systems (BMOSs)	1

Study	Year	Study Type (RCT =1, NRSI = 2, cRCT = 3)	Control	Intervention	Complexity of Intervention (Simple = 1, Complex = 2)
McEvoy AW, James OF. The effect of a dietary supplement (build-up) on nutritional status in hospitalized elderly patients. *Hum Nutr Appl Nutr* 1982;36(5):374-6.	1982	1	1. Standard care	1. ONS	1
Miller MD, Crotty M, Whitehead C, Bannerman E, Daniels LA. Nutritional supplementation and resistance training in nutritionally at risk older adults following lower limb fracture: a randomized controlled trial. *Clin Rehabil* 2006 Apr;20(4):311-23.	2006	1	1. Attention control plus usual care and general nutrition and exercise advice	1. Group I: ONS (6.3 kJ/mL) for six weeks<br>2. Group II: tri-weekly resistance training for 12 weeks<br>3. Group III: combined treatment for 24 weeks	2
Milte R, Miller MD, Crotty M, Mackintosh S, Thomas S, Cameron ID, et al. Cost-Effectiveness of Individualized Nutrition and Exercise Therapy for Rehabilitation Following Hip Fracture. *J Rehabil Med.* 2016;48(4):378-85.	2016	1	1. Attention control for 6 months.	1. A six-month individualized nutrition and exercise program was implemented for each participant.<br>2. The dietary component involved tailored counseling on meal timing, size, and frequency, as well as recommendations for nutrient-rich foods and recipes.<br>3. Referrals to community meal programs and provision of commercial oral nutritional supplements or protein powders were made as deemed appropriate.	2

**Study**	**Year**	**Study Type (RCT =1, NRSI = 2, cRCT = 3)**	**Control**	**Intervention**	**Complexity of Intervention (Simple = 1, Complex = 2)**
Munk T, et al. Positive effect of protein-supplemented hospital food on protein intake in patients at nutritional risk: a randomised controlled trial. *J Hum Nutr Diet* 2014;27(2): 122-32.	2014	1	1. Standard hospital food service	1. Participants were provided with a targeted food concept featuring an a la carte menu of small, energy-dense dishes, supplemented with high-quality protein powder.<br>2. The dishes could be ordered via telephone by patients, ward staff, or research assistants.<br>3. The ordered dishes were presented and served by kitchen staff utilizing a “room service” approach.	1
Munk T, Seidelin W, Rosenbom E et al. (2013) A 24-h a la carte food service as support for patients at nutritional risk: a pilot study. *J Hum Nutr Diet* 26, 268–275.	2013	2	NA	1. A “room service” approach was employed where dishes could be ordered by patients, ward staff, or research assistants from 07:00 to 20:00, seven days a week, with delivery guaranteed within 20 minutes of placing an order.<br>2. To ensure uninterrupted nutritional intake, especially during weekends with reduced ward staff, patients had the option to place orders 48 hours in advance.<br>3. The offered dishes were energy- and protein-enriched, fortified with a high-quality milk protein powder, and served in addition to the standard diet. These dishes contained 6.1-11.5 g of protein and 2.5-19.8 kJ/g of energy.	2

Study	Year	Study Type (RCT =1, NRSI = 2, cRCT = 3)	Control	Intervention	Complexity of Intervention (Simple = 1, Complex = 2)
Myint, M. W. W., Wu, J., Wong, E., Chan, S. P., To, T. S. J., Chau, M. W.R., Au, K. S. D. (2013). Clinical benefits of oral nutritional supplementation for elderly hip fracture patients: A single blind randomised controlled trial. *Age and Ageing*, 42, 39–45. https ://doi.org/10.1093/ageing/afs078	2013	1	1. Standard hospital diet	1. ONS + diet (n=65) for a maximum duration of 4 weeks	1
Neelemaat F, Bosmans JE, Thijs A, Seidell JC, van Bokhorstde van der Schueren MA. Post-discharge nutritional support in malnourished elderly individuals improves functional limitations. *Journal of the American Medical Directors Association* 2011;12(4):295-301	2011	1	1. Standard care treating physician may prescribe supplements.	1. Participants received standardized nutritional support from hospital admission until 3 months post-discharge, beginning with an energy- and protein-enriched diet during their in-hospital stay.<br>2. Nutritional supplementation included two servings of supplements daily, with 100% of patients initially on supplements and 84% continuing at the 3-month mark, alongside 400 IU vitamin D3 and 500 mg calcium per day.<br>3. After discharge, patients received bi-weekly phone counseling by a dietitian for a total of six sessions.	2
Niccoli S, Kolobov A, Bon T, Rafilovich S, Munro H, Tanner K, Pearson T, Lees SJ. Whey Protein supplementation Improves Rehabilitation Outcomes in Hospitalized Geriatric Patients: A Double Blinded, Randomized Controlled Trial. *J Nutr Gerontol Geriatr*. 2017 Oct-Dec;36(4):149-165.	2017	1	1. Received hot cereal and milk products without the whey protein.	1. Participants were given an oral dietary product containing a total of 24 g of whey protein per day, in addition to their usual diet.<br>2. The whey protein supplement (WPS) was incorporated into the participants' daily meals: 9 g mixed into hot cereal at breakfast and 7.5 g per drink at lunch and dinner.	1

**Study**	**Year**	**Study Type (RCT =1, NRSI = 2, cRCT = 3)**	**Control**	**Intervention**	**Complexity of Intervention (Simple = 1, Complex = 2)**
Olofsson, B, Stenvall, M, Lundstrom, M, Svensson, O, Gustafson, Y., 2007. Malnutrition in hip fracture patients: an intervention study. *J. Clin. Nurs*. 16 (11), 2027–2038. doi: 10.1111/j.1365-2702.2006.01864.x .	2007	1	1. Standard hospital diet	1. A nutritional journal was used to identify nutritional deficiencies, and protein-enriched meals were provided for a minimum of four days postoperatively.<br>2. At least two nutritional and protein drinks were administered daily throughout the hospitalization period.<br>3. Additional factors affecting the patient's nutritional status were also identified and managed.	2
Pedersen PU, Tewes M, Bjerrum M. Implementing nutritional guidelines the effect of systematic training for nurse nutrition practitioners. *Scand J Caring Sci* 2012; 26(1): 178-85.	2012	2	NA	1. Nurses underwent comprehensive training featuring five modules, each lasting 3–4 days.<br>2. Training modules included a blend of nutritional education and patient involvement in nutritional care, along with implementation strategies and change theories.<br>3. Each training day consisted of six hours of instruction.	2
Persson M, Hytter-Landahl A, Brismar K, Cederholm T. Nutritional Supplementation and Dietary Advice in Geriatric Patients at Risk of Malnutrition. *Clin Nutr.* 2007;26(2):216-24.	2007	1	1. Given brief written dietary advice (n=25)	1. Participants received two dietary counseling sessions by a dietitian: one before hospital discharge and another one week after.<br>2. All participants were prescribed 1-2 servings of nutritional supplements along with a daily multivitamin.<br>3. Dietitian maintained phone contact at three specific time-points: 1–2 weeks post-discharge, mid-study, and one week prior to follow-up.	2

Study	Year	Study Type (RCT =1, NRSI = 2, cRCT = 3)	Control	Intervention	Complexity of Intervention (Simple = 1, Complex = 2)
Porter J, Haines T, Truby H. The efficacy of Protected Mealtimes in hospitalised patients: a stepped wedge cluster randomised controlled trial. *BMC Med.* 2017;15(1):25. doi:10.1186/s12916-017-0780-1	2017	3	1. Standard care	1. The translation project employed four primary intervention approaches: education, restrictions, environmental restructuring, and enablement.<br>2. A variety of strategies were collaboratively developed with relevant staff to facilitate optimal implementation of protected mealtimes.<br>3. The aim was to create a multi-faceted approach that could address various challenges and ensure successful implementation.	2
Potter JM, Roberts MA, McColl JH, Reilly JJ. Protein energy supplements in unwell elderly patients - a randomized controlled trial. *JPEN Journal of Parenteral and Enteral Nutrition* 2001;25(6):323-9.	2000	1	1. Standard care	1. 1.5 kcal/mL supplement aimed to provide 22.5g protein and 540 kcal energy per day.<br>2. Administered thrice daily in 120 mL doses. Initiated within 48 hours of admission. 2. Nurses observed and recorded compliance during supplement administration, as with other medications.<br>3. Supplementation continued until discharge, death, or transfer to long-term care.	1
Poulsen I, Vendel Petersen H, Rahm Hallberg I, Schroll M. Lack of nutritional and functional effects of nutritional supervision by nurses: a quasi-experimental study in geriatric patients. *Scand J Food Nutr*. 2007;51(1):6-12. doi:10.1080/17482970701256245	2007	2	NA	1. Nutrition assessment incorporated into nursing plans.<br>2. Three-day dietary tracking by nurses.<br>3. Nurses trained via 90-min lectures.	2

Study	Year	Study Type (RCT =1, NRSI = 2, cRCT = 3)	Control	Intervention	Complexity of Intervention (Simple = 1, Complex = 2)
Rana SK, Bray J, Menzies-Gow N, Jameson J, James JJP, Frost P, et al. Short term benefits of post-operative oral dietary supplements in surgical patients. *Clinical Nutrition* 1992;11(6):337-44. [PUBMED: 16840018]	1992	1	1. Standard diet	1. Standard hospital diet supplemented ad libitum with a ONS (1.5kcal/ml)	1
Roberts HC, De Wet S, Porter K, Rood G, Diaper N, Robison J, et al. The feasibility and acceptability of training volunteer mealtime assistants to help older acute hospital inpatients: the Southampton Mealtime Assistance Study. *J Clin Nurs* 2014;23(21-22):3240-9.	2014	2	NA	1. Volunteers were trained in half-day sessions, designed by a speech and language therapist (SALT) and a dietitian.<br>2. Training was conducted in groups of up to eight volunteers, spanning seven different half-day sessions.<br>3. Post-training, each volunteer's competency was assessed by the speech and language therapist during a mealtime, approximately lasting 30 minutes.	2
Roberts HC, Pilgrim AL, Jameson KA, Cooper C, Sayer AA, Robinson S. The impact of trained volunteer mealtime assistants on the dietary intake of older female in-patients: the Southampton Mealtime Assistance Study. *J Nutr Health Aging* 2017;21(3):320-8.	2017	2	NA	1. A comprehensive training program for volunteers was designed by a multidisciplinary team comprising a speech and language therapist and a dietitian.<br>2. The training was segmented into seven half-day sessions, with each session accommodating groups of up to eight volunteers.<br>3. Following the training, a formal competency assessment was conducted for each volunteer by the speech and language therapist, focusing on their aptitude in fulfilling the roles and	2

Study	Year	Study Type (RCT =1, NRSI = 2, cRCT = 3)	Control	Intervention	Complexity of Intervention (Simple = 1, Complex = 2)
				responsibilities of a mealtime assistant. The evaluation lasted approximately 30 minutes per volunteer.	
Robinson S, Clump D, Weitzel T, Henderson L, Lee K, Schwartz C, et al. The memorial meal mates: a program to improve nutrition in hospitalized older adults. *Geriatr Nurs* 2002;23(6):332-5.	2002	2	NA	1. The volunteers were provided a 3 hr in-service taught by an interdisciplinary team composed of four nurses, a dietitian, an ST, and an occupational therapist.	2
Robison J, Pilgrim AL, Rood G, Diaper N, Elia M, Jackson AA, et al. Can trained volunteers make a difference at mealtimes for older people in hospital? A qualitative study of the views and experience of nurses, patients, relatives and volunteers in the Southampton Mealtime Assistance Study. *Int J Older People Nurs* 2015;10(2):136-45.	2015	2	NA	1. Volunteers trained in seven half-day sessions developed by a swallowing therapist and dietitian	2

Study	Year	Study Type (RCT =1, NRSI = 2, cRCT = 3)	Control	Intervention	Complexity of Intervention (Simple = 1, Complex = 2)
Rondanelli M, Klersy C, Terracol G, Talluri J, Maugeri R, Guido D, Faliva MA, Solerte BS, Fioravanti M, Lukaski H, Perna S. Whey protein, amino acids, and vitamin D supplementation with physical activity increases fat-free mass and strength, functionality, and quality of life and decreases inflammation in sarcopenic elderly. *Am J Clin Nutr*. 2016 Mar;103(3):830-40.	2016	1	1. Placebo with concurrent with regular, controlled physical activity (n=61)	1. Supplementation with whey protein (22 g), essential amino acids (10.9 g, including 4 g leucine), and vitamin D [2.5 mg (100 IU)] concurrent with regular, controlled physical activity	2
Rufenacht U, et al. Nutritional counselling improves quality of life and nutrient intake in hospitalized undernourished patients. *Nutrition* 2010;26(1): 53-60.	2010	1	1. ONS only	1. Patients were individually counseled by a dietitian following standardized procedures equivalent to standard care, leading to the creation of personalized nutritional plans.<br>2. The nutritional plans included various interventions such as food enrichment with energy and/or protein, utilizing supplements like maltodextrin, oil, and powdered protein.<br>3. ONS + standard hospital meals	2
Ryan M, et al. Oral nutritional supplements differing in fat and carbohydrate content: effect on the appetite and food intake of undernourished elderly patients. *Clin Nutr* 2004;23(4):683-9.	2004	2	NA	1. ONS	1

Study	Year	Study Type (RCT =1, NRSI = 2, cRCT = 3)	Control	Intervention	Complexity of Intervention (Simple = 1, Complex = 2)
Salisbury LG, Merriweather JL, Walsh TS. The development and feasibility of a ward-based physiotherapy and nutritional rehabilitation package for people experiencing critical illness. *Clin Rehabil*. 2010;24(6):489-500.	2010	2	NA	1. The enhanced nutritional rehabilitation program featured assistance at mealtimes, meticulous monitoring of supplement delivery and consumption, and frequent food charts to track and ensure adequate oral intake.<br>2. Generic rehabilitation assistants conducted one to two daily visits to facilitate the dietary and therapeutic tasks, although the duration of each visit was unspecified.<br>3. To equip the generic rehabilitation assistants with the necessary competencies, discipline-specific training was provided. However, details about the duration and quality of this training were not specified.	2
Saudny-Unterberger H, Martin JG, Gray-Donald K. Impact of nutritional support on functional status during an acute exacerbation of chronic obstructive pulmonary disease. *Am J Respir Crit Care Med* 1997;156(3 Pt 1):794-9	1997	1	1. Standard diet (n=16)	1. In the supplemental feeding group, patients received additional oral supplements like Ensure, Ensure Plus, or a variety of puddings along with their regular hospital tray.<br>2. The objective was to assure a caloric intake of at least 1.5 times the resting energy expenditure (REE) for patients with a normal body mass index (BMI) ranging from 20 to 27.<br>3. For those with a BMI below 20, the caloric intake target was set at a higher level of at least 1.7 times the REE.	2

**Study**	**Year**	**Study Type (RCT =1, NRSI = 2, cRCT = 3)**	**Control**	**Intervention**	**Complexity of Intervention (Simple = 1, Complex = 2)**
Schuetz P, et al. Individualised nutritional support in medical inpatients at nutritional risk: a randomised clinical trial. *Lancet* 2019;393 (10188):2312-21. Kaegi-Braun, et al. 2020	2020	1	1. Provided with standard hospital food, no nutritional consultation, and no recommendation for additional nutritional support	1. An individualized nutritional plan was crafted by a dietitian for each patient, addressing elements like food preferences, food fortification, inter-meal snacks, and nutritional supplements.<br>2. In the hospital setting, 91% of the patients in the intervention group received oral nutritional supplements as part of their tailored nutritional care.<br>3. Upon discharge, 24% of the patients in the intervention group continued to receive oral nutritional supplements as part of their ongoing nutritional management.	2
Schultz TJ, Kitson AL, Soenen S, Long L, Shanks A, Wiechula R, et al. Does a Multidisciplinary Nutritional Intervention Prevent Nutritional Decline in Hospital Patients? A Stepped Wedge Randomised Cluster Trial. *e-SPEN Journal.* 2014;9(2):e84-e90.	2014	3	1. Standard care (n=135)	1. Facilitator pairs consisting of a nurse from one of the step's wards and a dietitian were assigned to lead the nutritional intervention.<br>2. These facilitator pairs were responsible for educating and training staff on the use of the Malnutrition Universal Screening Tool (MUST), thereby enhancing the quality of nutritional assessments.<br>3. Additionally, they collaborated with the hospital kitchen to implement the use of red trays for specific nutritional needs, facilitating more tailored feeding assistance.	2

Study	Year	Study Type (RCT =1, NRSI = 2, cRCT = 3)	Control	Intervention	Complexity of Intervention (Simple = 1, Complex = 2)
Sharan kumar VG, Pajanivel R, Boratne AV, Vimal Raj R. Impact of Dietary Counselling on the Nutritional Status and Quality of Life among Pulmonary Tuberculosis Patients - a Randomized Control Trial. *Indian Journal of Tuberculosis*. 2021.	2021	1	1. Patients were advised to take high protein diet, but the diet was not charted by the nutritionist.	1. A personalized dietary counseling approach was implemented, focusing on locally available and culturally acceptable foods that aligned with the patients' socio-economic status. This was facilitated through diet charts provided by the nutritionist.<br>2. Counseling was initiated at the beginning of each patient's treatment to ensure timely intervention and address any nutritional deficits or concerns.<br>3. Follow-up consultations were conducted via phone throughout the treatment duration, allowing for dynamic adjustments to the dietary plan as needed.	2
Sharma Y, Thompson CH, Kaambwa B, Shahi R, Hakendorf P, Miller M. Investigation of the Benefits of Early Malnutrition Screening with Telehealth Follow up in Elderly Acute Medical Admissions. *Qjm*. 2017;110(10):639-47.	2017	1	1. Standard care. Dietitian review occurs only if patients are referred by a healthcare professional. No dedicated outpatient follow-up after discharge. (n=46)	1. A multi-faceted nutritional intervention was deployed that included nutritional supplements, food fortification, and mid-meal snacks while patients were in the hospital. This was supplemented by dietary counseling provided by a dietitian to both patients and caregivers.<br>2. Ward-based staff were available to assist with meals as needed, and ongoing support was provided through monthly telephone calls from the dietitian for a duration of two months post-discharge. These calls aimed to reinforce adherence to the dietary regimen.<br>3. Compliance with the intervention was rigorously evaluated through a 24-hour self-reported dietary recall method.	2

Study	Year	Study Type (RCT =1, NRSI = 2, cRCT = 3)	Control	Intervention	Complexity of Intervention (Simple = 1, Complex = 2)
Shyu YI, Liang J, Tseng MY, Li HJ, Wu CC, Cheng HS, et al. Comprehensive care improves health outcomes among elderly Taiwanese patients with hip fracture. *J Gerontol A Biol Sci Med Sci* 2013 Feb;68(2):188-97.	2013	1	1. The usual care group, consisting of 99 participants, received an average of 1.89 (SD = 2.32) physical therapy sessions during their hospitalization period.<br>2. Typically, these patients were discharged from the hospital approximately 5–7 days post-surgery. They received varied discharge health education from their primary nurses, but no organized home rehabilitation was provided.<br>3. Post-discharge, the usual care group underwent clinical follow-ups at predetermined time points—specifically, at 1, 3, and 6 months—to assess their recovery and health status.	1. The comprehensive care group received one year of in-home rehabilitation, including three in-home physical therapy (PT) sessions in the first four months following hospital discharge. These sessions were strategically scheduled within the first week, third week, and third month post-discharge. An additional PT session was administered at the sixth-month mark.<br>2. Beyond the rehabilitation framework, the comprehensive care regimen also incorporated other holistic elements such as geriatric consultation, discharge planning with post-hospital services, and a structured rehabilitation program.<br>3. Additional facets of the comprehensive care model included specialized consultations in nutrition, depression management, and fall prevention, offering a multidisciplinary approach to patient care	2

Study	Year	Study Type (RCT =1, NRSI = 2, cRCT = 3)	Control	Intervention	Complexity of Intervention (Simple = 1, Complex = 2)
Singh NA, Quine S, Clemson LM, et al. Effects of high-intensity progressive resistance training and targeted multidisciplinary treatment of frailty on mortality and nursing home admissions after hip fracture: a randomized controlled trial. *J Am Med Dir Assoc* 2012;13:24–30.	2012	1	1. Standard care as offered for hip fracture in the area health service, including orthogeriatric care, rehabilitation service, other medical and allied health consultation as required, and physiotherapy.	1. The experimental group underwent high-intensity resistance training at 80% peak strength, supervised bi-weekly for 12 months in an outpatient clinic.<br>2. Training started 6-8 weeks post-fracture, following standard physiotherapy. Coordination was managed through weekly team meetings.<br>3. Participants received monthly home visits and phone calls from trainers, totaling 80 training sessions, 10 visits, and 10 calls over a year.	2
Söderström L, Rosenblad A, Bergkvist L, Frid H, Thors Adolfsson E. Dietary Advice and Oral Nutritional Supplements Do Not Increase Survival in Older Malnourished Adults: A Multicentre Randomised Controlled Trial. *Upsala Journal of Medical Sciences*. 2020;125(3):240-9.	2020	1	1. Standard care	1. Dietary counseling group: Patients received a single counseling session by a dietitian before discharge, with no supplements. Follow-ups at 1, 3, and 6 months via phone.<br>2. Supplement group: Patients were instructed to consume 1–2 supplement bottles daily. 100% compliance. Follow-ups at the same intervals.<br>3. Combined croup: Received both dietary counseling and 1-2 daily supplement bottles. Follow-up calls at 1, 3, and 6 months post-discharge.	2
Starke J, et al. Short-term individual nutritional care as part of routine clinical setting improves outcome and quality of life in malnourished medical patients. *Clin Nutr* 2011;30(2):194-201.	2011	1	1. Standard nutritional care, including the prescription of ONS and nutritional therapy prescribed by the physician independently	1. Comprehensive nutritional assessment: Participants received an individualized assessment to tailor their food supply.<br>2. Meal fortification: Regular meals were enhanced with maltodextrin, rapeseed oil, cream, and/or protein powder to meet nutritional needs.	2

Study	Year	Study Type (RCT =1, NRSI = 2, cRCT = 3)	Control	Intervention	Complexity of Intervention (Simple = 1, Complex = 2)
			according to the routine ward management.	3. Additional nutritional support: In-between snacks and oral nutritional supplements were provided to supplement dietary intake.	
Stelten S, Dekker IM, Ronday EM et al. (2015) Protein enriched "regular products" and their effect on protein intake in acute hospitalized older adults; a randomized controlled trial. *Clin Nutr* 34, 409–414	2015	1	1. Regular bread and regular drinking yoghurt ad libitum.	1. Protein-enriched bread (Carezzo bv®) and protein-enriched drinking yoghurt (Drinking yoghurt enriched with Fonterra Whey Protein Concentrate (WPC. 515) by Fonterra Europe®) ad libitum.	1
Terp R, Jacobsen KO, Kannegaard P, Larsen AM, Madsen OR, Noiesen E. A Nutritional Intervention Program Improves the Nutritional Status of Geriatric Patients at Nutritional Risk—a Randomized Controlled Trial. *Clinical Rehabilitation*. 2018;32(7):930-41.	2018	1	1. Standard care. The clinical dietitian was involved in the process if the patient had specific needs and gave dietary advices and prepared a dietary plan for nutrition intake while they were hospitalized.<br>2. At discharge, no follow-up was planned.	1. Individualized plans: Dietitian-crafted plans based on everyday food, potentially supplemented.<br>2. Post-discharge care: Dietary advice for after hospital release.<br>3. Follow-up: Three visits at 1, 4, and 8 weeks post-discharge, conducted by district nurse or healthcare assistant.	2

Study	Year	Study Type (RCT =1, NRSI = 2, cRCT = 3)	Control	Intervention	Complexity of Intervention (Simple = 1, Complex = 2)
Van den Berg GH, Lindeboom R, Van der Zwet WC. The effects of the administration of oral nutritional supplementation with medication rounds on the achievement of nutritional goals: a randomised controlled trial. *Clinical Nutrition* 2015; 34:15-19.	2015	1	1. Usual care group was offered 125 ml of ONS by nutrition assistants twice a day in between meals at 10 am and 3 pm (n=88).	1. Group I: 125 ml ONS twice daily at 12 and 17 o'clock, aligned with medication.<br>1. Group II: 63 ml ONS four times daily at 8, 12, 17, and 20 o'clock, also with medication.<br>2. Participant count: Both interventions involved 146 subjects.	1
Vázquez-Sánchez M, Valero-Cantero I, Carrión-Velasco Y, Castro-López P, Suárez-Cadenas E, Casals C. Applicability and Clinical Validity of Nursing Outcomes Classification in a Nursing Intervention of Nutritional Counseling for Patients with Malnutrition. *Int J Nurs Knowl*. 2019;30(3):168-72.	2019	1	1. Standard care	1. Patients underwent nutritional counseling by case manager nurses, which began during the hospital stay and lasted for six months.	1
Vermeeren MA, et al. Nutritional support in patients with chronic obstructive pulmonary disease during hospitalization for an acute exacerbation; a randomized controlled feasibility trial. *Clin Nutr* 2004;23(5): 1184-92.	2004	1	1. Placebo ONS	1. ONS 125 ml x 3 serves/d (Respifors, Nutricia, Zoetermeer, The Netherlands, 2.38 MJ/day, 20 energy% protein, 20 energy% fat, and 60 energy% carbohydrate)	1
Vlaming S, et al. Should the food intake of patients admitted to acute hospital services be routinely Supplemented? A randomized placebo controlled trial. *Clin Nutr* 2001;20(6):517-26.	2001	1	1. Placebo ONS	1. ONS 400 ml/d (Ensure Plus, Abbott Laboratories Ltd)	1

Study	Year	Study Type (RCT =1, NRSI = 2, cRCT = 3)	Control	Intervention	Complexity of Intervention (Simple = 1, Complex = 2)
Volkert D, et al. Nutritional support and functional status in undernourished geriatric patients during hospitalization and 6-month follow-up. *Aging Clin Exp Res* 1996;8(6):386-95.	1996	1	1. Standard hospital diet	1. In addition to the standard hospital diet, patients were offered two portions of a liquid nutritional supplement (200 mL soup in the midmorning, 200 mL sweet drink in the afternoon) daily.<br>2. Different brands with similar composition but different flavors were used in order to increase variety and acceptance	2
Walton K, Williams P, Bracks J, Zhang Q, Pond L, Smoothy R, et al. A volunteer feeding assistance program can improve dietary intakes of elderly patients - a Pilot Study. *Appetite* 2008;51(2):244-8.	2008	2	NA	1. 25 volunteers, specifically recruited and trained (time unspecified)<br>2. 45 min to assist two to three patients with their lunch meal.	2
Wong A, Burford S, Wyles CL, Mundy H, Sainsbury R. Evaluation of strategies to improve nutrition in people with dementia in an assessment unit. *J Nutr Health Aging* 2008;12(5):309-12	2008	2	NA	1. Providing a glass-door refrigerator filled with snacks and beverages to which patients have access 24 hr a day, seven days a week.<br>2. Playing soothing music at meal times	1
Wong SY, Lau EM, Lau WW, Lynn HS. Is dietary counselling effective in increasing dietary calcium, protein and energy intakes in patients with osteoporotic fractures? A randomised controlled clinical trial. *Journal of Human Nutrition & Dietietics* 2004;17(4):359-6	2004	1	1. 500 mg of calcium supplementation and an anti-resorptive agent for the treatment of osteoporosis.	1. 500 mg of calcium supplementation and an anti-resorptive agent for the treatment of osteoporosis.<br>2. A one-to-one 45-minute session with a dietitian. Each patient received education about the importance of adequate calcium and protein in their diet.	2

**Study**	**Year**	**Study Type (RCT =1, NRSI = 2, cRCT = 3)**	**Control**	**Intervention**	**Complexity of Intervention (Simple = 1, Complex = 2)**
			2. Given a printed pamphlet, on the prevention of osteoporosis.		
Wright L, Cotter D, Hickson M. The effectiveness of targeted feeding assistance to improve the nutritional intake of elderly dysphagic patients in hospital. *J Hum Nutr Diet* 2008;21(6):555-62.	2008	2	NA	1. One-week training program by an experienced dietitian and SALT on targeted assistance	2
Wyers CE, Reijven PL, Breedveld-Peters JJ, Denissen KF, Schotanus MG, van Dongen MC, et al. Efficacy of nutritional intervention in elderly after hip fracture: a multicenter randomized controlled trial. *Journals of Gerontology. Series A, Biological Sciences and Medical Sciences* 2018;73(10):1429-37.	2018	1	1. Standard care	1. Regular counseling by a trained study dietitian<br>2. ONS for 3 months post-surgery, started during hospitalization and continuing in the rehabilitation center and/or, if applicable, at home.<br>3. Throughout the study, a patient was supervised by the same dietitian. Dietary advice, based on individual energy and protein requirements, comprised an energy- and protein-rich diet as well as recommendations on choice, quantity, and timing of foods (400 mL per day) for 3 months.<br>4. The dietitian had ten contacts with each patient: two during hospitalization and eight thereafter (three face-to-face and five telephone calls).	2

Study	Year	Study Type (RCT =1, NRSI = 2, cRCT = 3)	Control	Intervention	Complexity of Intervention (Simple = 1, Complex = 2)
Yang PH, et al. Effect of nutritional intervention programs on nutritional status and readmission rate in malnourished older adults with pneumonia: a randomized control trial. *Int J Environ Res Publ Health* 2019;16(23).	2019	1	1. Standard ONS according to the Kaohsiung Chang Gung Memorial Hospital Nutrition Department	1. Individualized nutrition plan crafted by a dietitian, involving both patients and their caregivers. The post-discharge diet was taught with dietary advice.<br>2. Unspecified percentage received supplements as part of their dietary plan.<br>3. Post-discharge follow-up included phone calls to monitor nutritional status and adapt plans; the frequency of calls was not reported.	2
Yoshimura Y, Uchida K, Jeong S, Yamaga M. Effects of ONS on Muscle Mass and Activities of Daily Living in Elderly Rehabilitation Patients with Decreased Muscle Mass: A Randomized Controlled Trial. *J Nutr Health Aging*. 2016 Feb;20(2):185-91.	2016	1	1. Resistance training only	1. ONS (Resource PemPal Active 200kcal/10g protein) + resistance training	1
Young AM, Banks MD, Mudge AM. Improving nutrition care and intake for older hospital patients through system-level dietary and mealtime interventions. *Clin Nutr ESPEN*. 2018;24: 140-147.	2018	2	NA	1. Added 1.0 FTE assistant-in-nursing (AIN) to help with meal set-up, feeding, and encouragement during meals and snacks.<br>2. Quarterly multidisciplinary education sessions aimed at junior medical officers and allied health professionals, although the specifics of the training were not detailed.<br>3. An additional 0.5 FTE multi-professional allied health assistant (AHA) was implemented to handle tasks to mitigate functional decline, delirium, and malnutrition, including meal assistance. No other changes to staffing levels.	2

Study	Year	Study Type (RCT =1, NRSI = 2, cRCT = 3)	Control	Intervention	Complexity of Intervention (Simple = 1, Complex = 2)
Young, A.M., Mudge, A.M., Banks, M.D., Ross, L.J., Daniels, L., 2013. Encouraging, assisting and time to EAT: Improved nutritional intake for older medical patients receiving protected mealtimes and/or additional nursing feeding assistance. *Clin. Nutr*. 32, 543–549. doi:http://dx.doi.org/10.1016/j.clnu.2012.11.009	2013	2	NA	1. Whole-team approach with no staffing changes, but strategies were negotiated to prioritize mealtimes by minimizing non-urgent tasks and scheduling interruptions outside of mealtimes. 2. Comprehensive 25-hour clinician education program, inclusive of 6 hours for nursing, aimed at reinforcing the importance of patient nutritional intake. 3. Addition of one assistant-in-nursing staff member per ward to assist registered nurses and dietitians, specifically focused on monitoring plate waste and providing early nutrition support to "at-risk" patients.	2

HMB: β-hydroxy-β-methylbutyrate; ONS: Oral Nutritional Supplement; cRCT: Cluster Randomized Controlled Trials; RCT: Randomized Controlled Trial, NRSI: Non-Randomized Study of Intervention

Table S2. Sensitivity Analysis for Complex versus Simple Interventions. Logistic regression model using identified variables.

Coefficients

						Wald Test				95% Confidence interval (odds ratio scale)	
Model	Parameter	Estimate	Robust Standard Error	Odds Ratio	z	Wald Statistic	df	p	VS-MPR*	Lower bound	Upper bound
	(Intercept)	5.17	1.30	176.43	3.97	16.56	1	< .001	535.84	13.72	2268.67
	No. HCPs (Specialisation) Involved (n)	-1.13	0.34	0.32	-3.34	10.43	1	< .001	61.97	0.17	0.63
	Specific Interventions Employed (n)	-0.73	0.28	0.48	-2.65	6.65	1	8.09×10^{-3}	9.44	0.28	0.83
	No. of Intervention Areas Targeted (n)	-0.77	0.62	0.46	-1.23	2.06	1	0.22	1.11	0.14	1.58

Note. Complex coded as class 1.

* Vovk-Sellke Maximum *p* -Ratio: Based on the *p* -value, the maximum possible odds in favor of H_1 over H_0 equals $1/(-e\ p\ \log(p))$ for $p \leq .37$ (Sellke, Bayarri, & Berger, 2001).
